# Thyroid and Pseudothyroid Dysfunction as a Cause That is Promoting the Relapse of Benign Focal Thyroid Pathology

**DOI:** 10.25122/jml-2020-0130

**Published:** 2020

**Authors:** Nina Petrivna Tkachuk

**Affiliations:** Department of Surgery No. 1, Higher State Educational Establishment of Ukraine “Bukovinian State Medical University”, Chernivtsi, Ukraine

**Keywords:** Relapse risk, nodular goiter, benign focal pathology, thyroid gland

## Abstract

Several studies deal with learning causes stipulating nodular formations in the thyroid tissue, including those occurring against the ground of metabolic disorders of thyroid hormones. Our study’s objective was to determine the peculiarities of thyroid homeostasis disorders in patients suffering from benign nodular thyroid pathology with relapses of the disease and its relapse-free course. For this purpose, 96 female patients suffering from nodular thyroid pathology and 20 without thyroid pathology were examined. In the course of the study, the following were found in patients with benign focal thyroid pathology: disorders of the peripheral conversion of the thyroid hormones, compensatory activation of the hypothalamic-pituitary system evident in increased levels of the thyroid-stimulating hormone, ТSH/fT3 and ТSH/fT4 ratios, increasing titers of the anti-thyroid antibodies which can be hazardous for the risk of development of nodules or reflects the process of thyroid tissue damage; high level of thyroglobulin caused by an increased probability of relapse and rate of nodule growth, an increase of the thyroid gland volume associated with activation of the hypothalamic-pituitary system, increased antibodies titer and thyroid gland damage. Thus, changes of the examined indices in the blood can be used as prognostic markers concerning the relapse of nodule formation in the thyroid tissue.

## Introduction

The nodular degeneration of the thyroid is a common condition and, depending on the definition and type of imaging, it affects 20–76% of patients on a background of delicate interactions between genetic and environmental factors [1]. The majority of researchers indicate an annual world increase of patients with benign focal thyroid pathology, which will certainly maintain a significant number of primary surgical procedures, and implicitly, high relapse rates [2, 3]. One of the unsatisfactory results of surgical treatment of nodular and mixed forms of goiter is relapse occurring after surgery [4]. However, the methods that prevent the disease remain disputable. Nowadays, a full range of pre-surgical diagnostics of nodular formations in the thyroid gland is directed to determining their oncological risk [5-7]. Nevertheless, recommendations concerning an adequate spectrum of surgery in the case of nodular forms of goiter in terms of probable relapses are practically lacking in the literature. The priority to maintain healthy functionally-able thyroid parenchyma with maximum removal of pathologically changed one during the first surgery should remain indisputable and undeniable.

Nowadays, a number of studies deal with the investigation of causes stipulating the formation of nodular formations in the thyroid tissue, including those occurring against the ground of metabolic disorders of the thyroid hormones [8-20].

The following negative factors concerning an increased risk of nodules are found in the literature: decreased thyroid function (including cases with inadequate substitution therapy), activation of the hypothalamic-pituitary system with a tendency to thyroid-stimulating hormone (TSH) increase and decrease of the peripheral conversion of low-activity Т4 into active Т3, (low Т3 syndrome), as a rule, at the expense of comorbid diseases and pathological conditions [9-11].

## Material and Methods

The objective of our study was to determine the peculiarities of thyroid homeostasis disorders in patients suffering from benign nodular thyroid pathology with relapses of the disease and its relapse-free course.

To analyze probable causes of nodular thyroid pathology relapses, 96 patients with focal thyroid pathology (main groups) were examined. They were divided into the following groups: the first one included 30 female patients with postsurgical relapse of nodular (multinodular) euthyroid goiter; the second group included 30 female patients with nodular (multinodular) euthyroid goiter with the rapid growth of nodules confirmed by ultrasonography findings; the third group included 36 female patients with nodular (multinodular) goiter and slow growth of thyroid nodules diagnosed by means of ultrasound. Twenty females without thyroid pathology constituted the group of comparison.

Functional state and autoimmune aggression indices of the thyroid gland were determined by means of the immunochemical method with electrochemiluminescence detection (ECLIA).

To determine the functional state of the pituitary-thyroid gland system, fТ3/fТ4, ТSH/fТ4 coefficients, and the ТSH/fТ3 and thyroid index (ТІ) ratios were calculated:


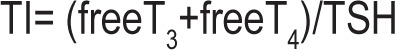


The peripheral activity of the thyroid hormones was assessed by means of the total thyroid index (TTI):


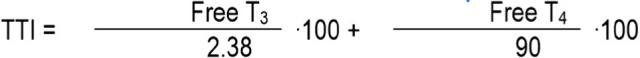


The results of the study obtained were statistically processed by means of the electronic tables, using Microsoft® Office Excel and the PAST (Paleontological Statistics Software Package for Education and Data Analysis) software for statistical processing. Differences between the study groups were checked using Student’s t-test. The probability of differences (р) was considered to be statistically reliable at р≤0.05.

## Results

In the process of the investigation, 14.8% of patients from the main group were found to have lower values of free T3 (fТ3) in comparison with the reference values, while free T4 (fТ4) and TSH were within the normal range, which can be considered as a disorder of the peripheral conversion of the thyroid hormone with development of a “low Т3 syndrome”.

In all the subgroups of the main group, the TSH level was found to be statistically higher compared to the control group. Thus, in the group with nodular goiter relapse, it was 2.36 times greater, 1.85 times in the group with nodular goiter with rapid growth, and 1.46 times in the group with nodular goiter with slow growth compared to the group of healthy individuals (р<0.05) (Table 1). The highest value of TSH was found in the group with nodular goiter relapse. The data appeared to be reliable concerning the groups with nodular goiter with rapid and slow growth (26.8% and 61.2%, respectively) (р<0.05). TSH level in the blood serum of individuals with nodular goiter and rapid growth appeared to be 27.1% (р<0.05), significantly higher than that of individuals with nodular goiter and slow growth.

**Table 1:** Functional values of the thyroid gland state at the point of surgery in the examined patients.

	Relapse of nodular goiter (I) n=30	Nodular goiter with rapid growth (II) n=30	Nodular goiter with slow growth (III) n=36	Without thyroid pathology n=20
**TSH, mIU/L**	3.45 ± 0.25 p* <0.05 p** <0.05 p*** <0.05	2.72 ± 0.24 p** <0.05 p*** <0.001	2.14 ± 0.17 p*** <0.05	1.46 ± 0.19
**fT_3_, pg/ml**	2.71 ± 0.24 p* <0.05 p** <0.05 p*** <0.05	2.96 ± 0.12 p** <0.05 p*** <0.05	3.54 ± 0.23 p*** >0.05	3.98 ± 0.291
**fT_4_, pg/ml**	1.09 ± 0.32 p* >0.05 p** >0.05 p*** >0.05	1.01 ± 0.24 p** >0.05 p*** >0.05	1.10 ± 0.33 p*** >0.05	0.98 ± 0.08
**Thyroid Index (TI) **	1.07 ± 0.09 p* <0.05 p** <0.05 Р*** <0.01	1.45 ± 0.10 p** <0.05 p*** <0.05	2.17 ± 0.13 p*** <0.05	3.49 ± 0.37
**Total Thyroid Index (TTI) **	115.01 ± 8.36 p* >0.05 p** <0.001 p*** <0.001	125.5 ± 8.15 p** <0.05 p*** <0.01	149.9 ± 5.11 p*** <0.05	168.8 ± 6.60
**TSH/fT_4_**	3.18 ± 0.15 p* >0.05 p** <0.001 p*** <0.001	2.75 ± 0.18 p** <0.05 p*** <0.001	1.94 ± 0.09 p*** <0.001	1.49 ± 0.09
**TSH/fT_3_**	1.27 ± 0.14 p* <0.05 p** <0.05 p*** <0.05	0.91 ± 0.09 p** <0.05 p*** <0.05	0.61 ± 0.08 p*** <0.05	0.37 ± 0.03
**fT_3_/fT_4_**	2.49 ± 0.13 Р* <0.05 Р** <0.05 Р*** <0.05	2.99 ± 0.16 Р** <0.05 Р*** <0.05	3.21 ± 0.19 Р*** <0.05	4.09 ± 0.14
**Thyroperoxidase (TPO) antibodies at the point of surgery, mIU/ml**	61.4 ± 4.14 Р* >0.05 Р** <0.001 Р*** <0.001	58.3 ± 6.21 Р** <0.05 Р*** <0.05	42.4 ± 3.22 Р*** <0.050	24.1 ± 4.18
**Anti-thyroglobulin autoantibodies at the point of surgery, mIU/ml **	116.3 ± 3.12 Р* <0.05 Р** <0.05 Р*** <0.05	97.4 ± 5.09 Р** < 0.05 Р*** <0.05	81.6 ± 5.30 Р*** <0.05	48.1 ± 3.08
**Thyroid hormones (TH) at the point of surgery, ng/ml**	92.1 ± 5.14 Р* <0.05 Р** <0.05 Р*** <0.001	86.2 ± 6.12 Р** >0.05 Р*** <0.001	76.3 ± 3.15 Р*** <0.001	12.4 ± 2.11

Note: p* – probability of divergence with patients suffering from nodular goiter with rapid growth; p** - probability of divergence with patients suffering from nodular goiter with slow growth; p*** - probability of divergence with volunteers without thyroid pathology.

A significantly lower level of fТ3 (46.9%) was found in the group of individuals with nodular goiter relapse, and nodular goiter with rapid growth (34.5%, р<0.05) in comparison with the group of healthy individuals. A statistically significant decrease in serum fТ3 levels was obtained in the group of individuals with nodular goiter relapse and the group with nodular goiter with fast growth in comparison with individuals suffering from nodular goiter with slow growth: 30.6% and 19.6%, respectively (р<0.05).

In all the individuals from the main group, the fТ3/fТ4 ratio decreased significantly in the nodular goiter relapse group by 63.4%, in the nodular goiter with rapid growth group by 36.8%, and by 27.4% in the nodular goiter with slow growth group in comparison with the group of healthy individuals (р<0.05). In the groups of individuals with nodular goiter relapse and nodular goiter with rapid growth, the values of this index were statistically significantly lower concerning the group of individuals with nodular goiter with slow growth: 28.9% and 7.4%, respectively (р<0.05).

The TSH/fТ3 ratio appeared to be statistically significantly higher in all the individuals of the main group: 3.4 times higher in patients with nodular goiter relapse, 2.5 times higher in nodular goiter with rapid growth, and 1.7 times higher in nodular goiter with slow growth (р<0.05). In the groups of individuals with nodular goiter relapse and nodular goiter with rapid growth, the value of this index was statistically considerably lower compared to the group of individuals with nodular goiter and slow growth: 2.1 and 1.4 times, respectively (р<0.05).

The TSH/fТ4 ratio was significantly higher (84.5%) in patients with nodular goiter and rapid growth, in those with nodular goiter and slow growth – 30.2% compared to the group of healthy individuals (р<0.05). In the groups of individuals with nodular goiter relapse and nodular goiter with rapid growth, the value of this index was statistically higher compared to the group of individuals with nodular goiter and slow growth: 63.9% and 41.8%, respectively (р<0.05).

A significant decrease by 3.26 times of the thyroid index (ТІ) was found in individuals with nodular goiter relapse, by 2.4 times in the nodular goiter with rapid growth group and by 1.6 times in the nodular goiter with slow growth group compared to the group of healthy individuals. In the groups of individuals having nodular goiter relapse and nodular goiter with rapid growth, the value of this index was statistically considerably lower compared to the group of individuals with nodular goiter and slow growth: 2.02 and 1.5 times, respectively (р<0.05).

Total Thyroid Index (TТІ) values reflecting changes of the peripheral conversion of the thyroid hormones was 34.5% lower in the group of individuals suffering from nodular goiter with rapid growth, and 12.6% in the group with nodular goiter and slow growth compared to the control group (р<0.05). In the groups of individuals having nodular goiter relapse and nodular goiter with rapid growth, TТІ was statistically lower compared to the group of individuals with nodular goiter and slow growth: 30.3% and 19.5%, respectively (р<0.05).

Titers of anti-thyroid antibodies were significantly higher in all the individuals of the main group as compared with the control group (р<0.05).

Thus, the titer of anti-thyroglobulin autoantibodies appeared to be 2.41 times higher in individuals with nodular goiter relapse, 2 times higher in the nodular goiter with rapid growth group and 1.69 times higher in patients with nodular goiter with slow growth, statistically higher in comparison with the control group (р<0.05). In the groups of individuals having nodular goiter relapse and nodular goiter with rapid growth, the titer of anti-thyroglobulin autoantibodies was statistically lower compared to the group of individuals with nodular goiter and slow growth: 42.5% and 19.4%, respectively (р<0,05).

Significantly higher titers of thyroperoxidase (TPO) antibodies were obtained in individuals with rapid growth by 2.6 times and with nodular goiter and slow growth by 2.4 times, compared with the control group (р<0.05). In the groups of individuals having nodular goiter relapse and nodular goiter with rapid growth, TPO antibodies titer was statistically lower compared to the group of individuals with nodular goiter and slow growth: 44.8% and 37.5%, respectively (р<0.05).

Тhyroid hormones (TH) values were significantly higher in all the individuals of the main group compared to the control group: by 7.4 times in those with nodular goiter relapse, by 6.9 times in patients with nodular goiter with rapid growth and by 6.2 times in those with nodular goiter with slow growth (р<0.05). In the groups of individuals with nodular goiter relapse and nodular goiter with rapid growth, TH levels were significantly lower compared to the group of individuals with nodular goiter and slow growth: 20.7% and 12.9%, respectively (р<0.05).

According to the ultrasound findings, the volume of the thyroid gland was reliably higher in all the subgroups of the main group in comparison with the group of healthy individuals (р<0.05) (Table 2). It appeared to be the highest in patients with nodular goiter relapse. In the groups of individuals with nodular goiter relapse and nodular goiter with rapid growth, it was reliably higher compared to those with concerning those with nodular goiter and slow growth – 61.5% and 44.3%, respectively (р<0.05).

**Table 2:** Parameters of the thyroid gland volume according to ultrasound findings at the point of surgery of the examined patients.

	Relapse of nodular goiter n=30	Nodular goiter with rapid growth n=30	Nodular goiter with slow growth n=36	Without thyroid pathology n=20
Thyroid gland volume according to ultrasound at the point of surgery*	31.03 ± 2.19 p* <0.05 p** <0.01 p*** <0.001	24.74 ± 2.13 p** <0.05 p*** <0.001	19.22 ± 1.68 p*** <0.001	11.62 ± 1.47

Note: p* – probability of divergence with patients suffering from nodular goiter with rapid growth; p** - probability of divergence with patients suffering from nodular goiter with slow growth; p*** - probability of divergence with volunteers without thyroid pathology. * For patients with goiter relapse the volume of the thyroid gland according to ultrasound before the latter surgery was considered.

## Discussion

The data obtained indicate the activation of the hypothalamic-pituitary system resulting in triggering the mechanisms promoting the formation of nodules, including the activation of growth factors such as the insulin-like growth factor. These changes could be stipulated by disorders of the peripheral conversion of the thyroid hormones, which was manifested in the examined patients by decreased fТ3/fТ4 ratio and TТІ. Disorders of the peripheral conversion of the thyroid hormones with development of the “low Т3 syndrome” can be stipulated by comorbid pathology associated with decreased activity of deiodinase resulting from a lesion of the organs where they are synthesized or inhibition of their activity as a consequence of increased expression of cytokines, or against the ground of selenium insufficiency (directly contained in the active center of these enzymes). This disorder is known to occur in approximately 70% of hospitalized patients [10-11].

The increase of titers of anti-thyroid antibodies in the main group can be both the first cause promoting the growth of nodules and the result of pathological processes in the thyroid tissue. In turn, thyroglobulin levels increase in the individuals from the main group can be stipulated by a stimulating effect of TSH, lesion of the thyroid gland by an inflammatory process, high risk of relapse of benign formations (evidenced by the highest values of the index among the individuals from the first group) [13].

Positive correlations of an average force between the thyroid gland volume and titers of antithyroglobulin autoantibodies (r = 0.365, р<0.05), thyroperoxidase (TPO) antibodies (r = 0.389, р<0.05), TSH (r = 0.428, р<0.05), ТH (r = 0.478, р<0.05) are found as well as negative ones with fТ3/fТ4 ratio (r = - 0.334, р<0.05).

The correlation analysis conducted has found certain relations available between the indices of thyroid homeostasis, titers of anti-thyroid autoantibodies, TH levels, and volume of the thyroid gland.

## Conclusions

Patients with benign focal thyroid pathology develop disorders of the peripheral conversion of the thyroid hormones, which is manifested by a decreased serum level of free triiodothyronine, an increased level of free thyroxin, a decreased coefficient of free triiodothyronine/free thyroxin and total thyroid index. Moreover, a compensatory activation of the hypothalamic-pituitary system was registered, evidenced by an increased level of the thyrotrophic hormone, the thyrotrophic hormone/free triiodothyronine, and thyrotrophic hormone/free thyroxin ratios.

Increased titers levels of anti-thyroid antibodies were found in patients with benign focal thyroid pathology, which can increase the risk of development of nodules or reflect the processes of tissue damage in the thyroid gland.

A high level of thyroglobulin is caused by an increased probability of relapse and the rate of nodule growth. An increased volume of the thyroid gland is associated with activation of the hypothalamic-pituitary system, disorders of the peripheral conversion of the thyroid hormones, increased titers of antibodies, and thyroid gland lesions.

Therefore, the above indices can be used as prognostic markers concerning the relapse of nodule formation in the thyroid gland tissue.

## Conflicts of Interest 

The authors declare that there is no conflict of interest.
